# Work-Related Accidents and Sharp Injuries in Paramedics—Illustrated with an Example of a Multi-Specialist Hospital, Located in Central Poland

**DOI:** 10.3390/ijerph14080901

**Published:** 2017-08-10

**Authors:** Anna Garus-Pakowska, Franciszek Szatko, Magdalena Ulrichs

**Affiliations:** 1 Department of Hygiene and Health Promotion, Medical University of Lodz, 90-647 Lodz, Poland; franciszek.szatko@umed.lodz.pl; 2 Department of Econometrics, University of Lodz, 90-214 Lodz, Poland; magdalena.ulrichs@uni.lodz.pl

**Keywords:** paramedics, accidents, sharp injuries, work, occupational exposure

## Abstract

(1) Background: An analysis of work-related accidents in paramedics in Poland by presenting the model and trend of accidents, accident rates and by identifying causes and results of accidents; (2) Methods: A retrospective analysis of medical documentation regarding work-related accidents in a multi-specialist hospital, located in central Poland, in the period 2005–2015. The study group included paramedics who had an accident while being on duty; (3) Results: According to hospital records, 88 paramedics were involved in 390 accidents and 265 injuries caused by sharp instruments. The annual accident rate was 5.34/100 employed paramedics. Most of the accidents occurred at night. The most common reason for the accident was careless behaviour of the paramedic, which resulted in joint sprains and dislocations. Injuries accounted for a huge portion of the total number of events. As many as 45% of injuries were not officially recorded; (4) Conclusion: High rates of work-related accidents and injuries caused by sharp instruments in paramedics are a serious public health problem. Further studies should be conducted in order to identify risk factors of accidents, particularly injuries, and to implement preventative programmes, aiming to minimise rates of occupational hazards for paramedics.

## 1. Introduction

According to legal regulations introduced in Poland in 2002 [1], a work-related accident is a sudden event, due to an external cause, which results in a trauma or death, occurring during professional work. The law also defines the terms: a fatal work-related accident (the person died within a period of six months following the accident), a serious work-related accident (the person sustained serious bodily injury, fell victim to an incurable or life-threatening disease, became unable to do his/her professional work or his/her body became permanently disfigured) and a collective work-related accident (at least two employees fell victim to the same work-related accident).

An accident can be identified as a work-related accident if four requirements are met. They are the following:

1 The accident occurs suddenly [2]; the sudden occurrence is related to an external cause and not as a consequence of the occupational accident. The effects of the sudden accident do not have to occur straight afterwards [3].
2 The accident occurs due to external causes [4,5].
3 The accident is work-related [6].
4 The work-related accident may result in: temporary inability to work, permanent or long-term detriment to health, death of the employee.

If any of the above requirements are not met, a particular event cannot be classified as a work-related accident [7].

In 2013, the Minister of Health implemented a regulation [8] under which each injury caused by a sharp instrument is defined as a work-related accident. According to regulations, each such incident should be reported to a person responsible for keeping the register of work-related accidents. The quoted regulation in Poland is a transposition of the Directive of the Council of the European Union 2010/32/UE as of 10 May 2010, on executing a framework contract regarding prevention against injuries caused by sharp instruments in hospitals and healthcare centres, conducted by HOSPEEM (European Hospital and Healthcare Employers’ Association) and EPSU (European Federation of Public Service Unions) [9].

In recent years in Poland, the number of work-related accidents does not display any particular tendency. In 2013, 88,267 people were involved in work-related accidents [10]. In 2015, the number decreased to 87,622 [11], whereas in 2016 the number was 87,886 [12].

Each year, about 10% of work-related accidents constitute events classified under the healthcare and social care sectors [10,11,12]. In this group, females make up the substantial majority (80% in 2016) and midwives and nurses are most exposed to the risk of being involved in an accident. Unfortunately, registers of work-related accidents contain no separate information on the number of accidents in paramedics, who due to the specificity of their work—involving the provision of medical services in hazardous or life threatening circumstances—or due to the workplace (any place where such services have to be provided), are exposed to many factors which directly or indirectly increase the risk of work-related accidents. In the United States, emergency medical service workers have been shown to be more at risk of accident than workers in other economic sectors [13]. Data on medical rescuers show that they are a vocational group with a higher risk of occupational accidents. For example, in Australia this risk was six times higher than in other occupational groups [14], and in the United States the rate of occupational fatalities among paramedics was more than twice that of all other occupations [15].

Environmental factors related to the paramedic profession which might cause accidents include:

- Colliding with immovable objects—mainly ambulance equipment and objects located where first aid is given—resulting in contusions, cuts and bodily injuries (getting trapped, fractures).
- Sharp instruments and objects (needles, knives) causing cuts and infections.
- Slippery surfaces, on which one can fall down (as a result of haste, carelessness, dim light or lack of light); the falls might result in contusions, fractures and other bodily injuries.
- Falls from a height and falls while going down stairs, particularly while transporting e.g., transporting a patient in restricted visibility; the falls might result in bodily injuries, mainly head and spine traumas.
- Traffic accidents, leading to bodily injuries or death [16].

As it has been mentioned before, both work-related accidents and injuries caused by sharp instruments have to be registered. Unfortunately, in Poland there are still no accurate data on the number of instances of accidental breaches of tissue integrity. The data which are available are only estimates (e.g., 1480 accidents which happened while handling sharp tools were officially reported as work-related accidents [17], whereas it is estimated that the number of sharp injuries might be as high as 37,000 [18]. In the United States, there are 385,000 injuries per year among hospital workers [19].

Like falls and accidents which might contribute to the detriment of the health of the injured paramedic, exposure to infected material might also result in a public health hazard. While performing life-saving procedures/giving first aid, the paramedic might also be a source of infection for a patient. The most disastrous consequences include blood-borne infections [20].

The main aim of the study was to analyse the work-related accident rate in paramedics based on a register held in a selected multi-specialist hospital in central Poland. The detailed aims included:

(1) Analysis of the accident rate in paramedics employed in two different organizational units of the selected hospital.
(2) Analysis of the needlestick injuries/sharp injuries in paramedics.
(3) Estimation of the accident and injury trends in the period 2005–2015.
(4) Analysis of causes and effects of the registered work-related accidents.

## 2. Materials and Methods

The study presents information on work-related accidents experienced by paramedics employed in two units, i.e., in the Hospital Emergency Department (HED) and Emergency Medical Service (EMS) in the period 2005–2015, in one of the multi-specialist hospitals in central Poland. Data on the work-related accidents were obtained from unpublished hospital records—the authors used work-related statistical charts and the register of sharp injuries. Since the Emergency Medical Service was only incorporated into the hospital organization in 2013, these data come from archive documentation of an independent unit, i.e., from the Emergency Medical Service. The approximation of the number of employees employed in the EMS between 2005 and 2012 was made using a fixed average employment structure estimated on the basis of data from the years 2013–2015—the average share of EMS employees in the total employed in the hospital was about 10.10%, while the share of EMS rescuers in the analysed period was approximately 76.80%.

The data regard paramedics who were involved in individual or collective accidents. A work-related accident is identified as an event of any kind which affects a working paramedic (according to the classification of the Main Statistical Office in Poland).

For the purpose of the statistical analysis, the authors used the Statistica 12.0 (Statistica, Tulsa, OK, USA) and EViews 9.5 (IHS Global Inc, Irvine, CA, USA) programmes. They applied elements of a descriptive analysis for the evaluation of the accident and injury rates in paramedics. Accidents were evaluated by: the type of organizational unit, the age and the length of employment of the paramedic, the accident site, the time of day at which the accident occurred (duty hours—in the morning, in the afternoon, in the evening), the day the accident happened within a calendar year (winter vs. summer months) and the time of the accident following the beginning of duty. For the purpose of evaluation of a correlation between variables, the authors applied Pearson’s chi-square independence test. *p* ≤ 0.05 was adopted as statistically significant. In order to determine the degree of correlation between the measured variables, the authors calculated the Pearson’s contingency coefficient. The method of least squares (ordinary least squares estimator or OLS) was used to estimate the function of the linear trend. Accident and injury rates in paramedics who were involved in accidents in subsequent years were also calculated according to the following formula:

**Accident rate** = n_1_/N × 100

**Injury rate** = n_2_/N × 100

where: n_1_ is the number of accidents in paramedics; n_2_ is the number of injuries in paramedics; N is the number of paramedics at risk of accidents/injuries.

The head of the hospital and the Bioethics Committee of the Medical University of Lodz gave their consent for analysing the data beforehand, Document No. RNN /163/14/KB of 11.02.2014.

## 3. Results

### 3.1. Characteristics of the Hospital and the Study Group

The analysis of accidents was made in a multi-specialist hospital, located in an area of the Wielkopolska Region. The hospital consists of 25 wards (31 December 2015) and the average number of patients treated annually is 37,000. In 2015, the hospital employed 1113 medical personnel (the average number of medical staff in 2005–2015 was 1024; range: 860–1113). Paramedics are employed in two units. The first one is the Hospital Emergency Department (HED). It employs 65 medical personnel of whom 20 are paramedics (including 2 paramedics who are also driving licence holders). The other unit is the Emergency Medical Service (EMS), which employs 110 medical personnel. Of that number, there are 86 paramedics (including 41 paramedics who are also driving licence holders) (31 December 2015). On average, in the period 2005–2015, both units employed 81 medical staff (range: 77–89.5). Of that number, more than half were paramedics (mean = 47.4; range: 45.5–53).

In total, in the period 2003–2015, 390 work-related accidents and 265 sharp injuries were registered in the hospital. Of these numbers, 72 work-related accidents and 72 sharp injuries related to the employees of both organizational units. 53 paramedics were involved in the accidents and 35 were injured by sharp instruments (Table 1).

The injured paramedics were 23–57 years old (mean age: 35 years) and the mean length of employment was 10 years (ranging from 2 months to 29 years).

### 3.2. Analysis of Work-Related Accidents in Paramedics

The greatest numbers of accidents in which paramedics and paramedics holding a driving licence were involved were recorded in the hospital in 2010 and 2015 (eight paramedics in each year). In 2008, no paramedics were injured in an accident (Table 1). The reported data on the number of accidents among paramedics are characterized by a significant upward trend, on average about 0.45 cases per year (*p* = 0.053), but this is mainly due to the significant increase in the number of paramedics. The corresponding accident rate does not show a significant upward trend (mean non-significant decrease of about 0.16 (*p* = 0.41)).

In the study period, employees of an EMS, rather than those of an HED, were involved in work-related accidents (48 vs. 24). Similarly, EMS paramedics were involved in such accidents more frequently than HED paramedics (47 vs. 6). Table 2 presents the ratio of accidents which occurred in selected wards to accidents in which paramedics were involved.

On average, during the period 2005–2015, work-related accidents recorded in selected wards made up 6.15% and 12.31% of the total number of accidents in the Hospital Emergency Department and in the Emergency Medical Service, respectively. Every ninth accident took place in the Emergency Medical Service. With regards to the Hospital Emergency Department, accident victims were mostly nurses. On average, 25% of paramedics were involved in work-related accidents. Much more frequently, paramedics employed in the Emergency Medical Service were victims of such accidents (on average 95% of paramedics).

The accident rate for each 100 paramedics (at risk of work-related accident) ranged in the study period from 0 to 9.34 (mean rate: 5.34/100 paramedics).

In the HED, younger paramedics with a shorter length of employment were involved in accidents in comparison to the EMS (the HED: mean age—23 years, length of employment—5 years; the EMS: mean age—37.5 years, length of employment—11 years).

Paramedics working in the HED were involved in work-related accidents mainly in: surgical and internal medicine outpatient clinics, or a hall or a lift while transporting a patient. With regards to paramedics of the EMS, work-related accidents mostly occurred in an ambulance (slipping on steps, improperly gripping an instrument or a traffic accident). Accidents also happened in patients’ homes and in their yards.

Accidents in the HED mostly occurred in the summer months, during evening-night shifts (between 7:00 p.m. and 7:00 a.m.) and in the third hour following the beginning of duty. With regards to the EMS, work-related accidents also occurred mostly during the evening-night shift but in winter months (between October and March). The greatest number of accidents were recorded in the third, fourth and twelfth hour following the beginning of duty. From 2:00 a.m. onwards, the number of accidents gradually increased (Table 3).

Based on the chi-square independence test, there are no grounds for rejecting null hypotheses about the independence of tested variables: the time of year and the time of day for EMS and HED. The only significant differences which exist are between HED and EMS, and the seasons (Chi^2^Pearson = 5.40; *p* = 0.02). The results of the test may be underestimated due to the small size of the individual categories—the application of the Yates correction does not, however, change the overall conclusions.

The most common reason for work-related accidents was an improper reaction to an unexpected event or insufficient concentration. These were causes of 67.9% of work-related accidents (Table 4).

In no case did the authors observe a breach of health and safety procedures caused by the victim of the accident, either deliberately or resulting from gross negligence and no person involved in the accident had taken any psychoactive or psychotropic substances.

### 3.3. Results of Accidents

In the study period, serious or fatal accidents did not occur. In most cases, injuries involved joint sprain or dislocation affecting lower extremities (38%), upper extremities (32%) and neck, back and spine (17%). The number of sick leave days following the accident was on average 48 (the least 4 days, the most 189 days). Four injured people did not apply for sick leave. Due to a work-related accident, the injured were granted single compensations; the average amount was 475 US$ (min. 122 US$; max. 1592 US$). In most cases, detriment to health did not exceed 5%. In one case, the health loss was assessed to be 10%.

### 3.4. Needlestick Injuries/Sharp Injuries as Work-Related Accidents

Of the total number of work-related accidents which occurred in the hospital area (N = 655–390 work-related accidents and 265 sharp injuries), sharp injuries made up as much as 40% of cases. A similar percentage value (39.77%) was observed for injuries caused by sharp instruments in comparison with the total number of work-related accidents in paramedics. Among medical personnel of the HED, nurses rather than paramedics suffered from needlestick injuries (nurses: n = 26.79% and paramedics: n = 7.21%). In comparison to all hospital wards, needlestick injuries in the HED constituted 12%. The injury rate per 100 employed paramedics was from 0 to 43. In as many as 45% of cases (15 people) needlestick injuries were not reported to an officer of the Health and Safety Institute.

With regards to employees of the EMS, the majority of injuries involved paramedics (72%). The injury rate was 1.2–5.18/100 paramedics. Injuries sustained by employees of the EMS constituted 15% of the total number of hospital injuries (Table 5).

## 4. Discussion

All the events presented in the study were work-related accidents. They occurred suddenly while their victims were performing their professional duties, were caused by external reasons and contributed to the detriment of health. The cause of an accident is identified with any shortcomings and abnormalities which directly or indirectly led to its occurance. These causes could be associated with a material factor, general organization of work or work position as well as related to the employee himself/herself—for example his/her psychophysical condition or abnormal behaviour. In our study, the most common causes of accidents were either an improper reaction to an unexpected event e.g., a slippery road) or insufficient concentration as a result of haste (caused by the condition of the patient or a call to another accident site). Sustained injuries often resulted from physical overstrain following lifting patients. Overstrain of the skeletal and joint system is a consequence of transporting a patient, sometimes very heavy, in a difficult place e.g., a narrow staircase. Similarly to other authors, the authors of this study observed sprains and dislocations in most cases [21]. Spinal overstrain is a life-threatening condition and often leads to absenteeism, which is a financial burden for the state. In this study, the average number of a sick leave days was 48. Injuries of the locomotor system may turn into degenerative spine diseases, leading to other challenges associated with public health. In prophylaxis of such injuries we should remain particularly careful, observe rules regarding the load while lifting patients, use equipment which facilitates performing work (trolleys, lifts) and, if this is not possible, divide the load among a few employees. 

Results of studies conducted by other authors imply that the percentage of work-related accidents which involved ambulances is high [14,22]. As our study reveals, three work-related accidents were caused by motor vehicle collisions. While working with people, paramedics are faced with aggression, in most cases, this is verbal. Increasing aggression might result in assaulting a paramedic, which might in turn contribute to injuries. An analysis of the studied documentation revealed only one work-related accident caused by an aggressive person. However, professional literature shows that aggression is a huge problem which paramedics have to deal with while performing their occupational duties. As many as 87.5% of paramedics experience various forms of aggression in their workplace, which often results in injuries [22,23,24].

Risk factors, i.e., the time of day, lack of sleep and fatigue should be pointed out. Patterson et al estimated the relationship between the fatigue of emergency medical service workers and the risk of an accident [25]. In the presented study most of the cases occurred in the evening or at night (although it was not statistically significant). At this time, a human body demonstrates impaired attention and perception, which is associated with a natural circadian rhythm.

The accident trend did not significantly change with time. The trend related to sharp injuries did not change either. According to Polish regulations, all needlestick injuries/sharp injuries are considered work-related accidents. Hence, they are subject to registration. New legal regulations implemented in Poland were intended to provide more effective registration of such injuries. Unfortunately, they have appeared to be fairly ineffective. We should emphasize the high percentage of needlestick injuries in the total number of work-related accidents (40%) as well as the high percentage of needlestick injuries in accidents in which paramedics were involved (almost 40%). Results of the presented study indicate that paramedics are particularly exposed to the risk of injury, which correlates with the results of other studies [26]. Due to a high percentage of sharp injuries in all work-related accidents and a lack of comparable data in available registers, the authors stress that data gathered in hospital registers of injuries should be urgently evaluated and validated. It is supposed that many injuries are not reported to anybody or registered anywhere. This study revealed that information on 45% of injuries was not entered into an official register of injuries, and this is close to the results of other studies [27,28,29]. Reasons for such behavior require further studies. An in-depth analysis will help prepare programmes/guidelines/training for employees, which will in turn improve the control of injuries. Employees should be aware that reporting an injury is highly important and adjust their behaviour accordingly. Medical personnel are exposed to infection hazards but can also become a source of infection for patients [30,31]. Prevention of sharp injuries in paramedics might help them and future patients avoid infections, which would be of measurable benefit to public health.

### Limitations

The analysed data were found in unpublished hospital documentation. The information on work-related accidents was found in statistical accident charts, whereas data regarding injuries came from two different sources. Since 2013, in compliance with Polish regulations, a hospital is obliged to keep a register of injuries. Before 2013, data on injuries were not properly gathered. Another inconvenience was the incorporation of the Emergency Medical Service into the organization of the hospital. The authors gathered archival information on work-related accidents and injuries which had occurred in this organizational unit before 2013. However, it was a different unit then and information was gathered by someone who was not a hospital employee. Thus, this data may not correspond to the hospital data. We believe that the preparation and publication of epidemiological data is the first important step in presenting the real occupational risk facing paramedics, a procedure which we believe is essential.

## 5. Conclusions

(1) In the analyzed period, 53 paramedics suffered accidents at work and the average accident rate was 5.34 per 100 paramedics.
(2) Sharp injuries accounted for 40% of all accidents at work among paramedics. Attention is drawn to the high rate of non-compliance with the procedure for reporting a needlesticks.
(3) The number of accidents among paramedics is characterized by a significant upward trend, but this is caused mainly due to the significant increase in the number of paramedics. The number of sharp injuries in 2005–2015 has not changed significantly.
(4) The most common cause of the accident was an employee’s improper—but unconscious—behaviour. The most common effects of injuries were joint injuries.
(5) General conclusion: A high number of work-related accidents and sharp injuries in paramedics is a serious public health problem. Further studies are required in order to identify the risk factors of work-related accidents, particularly injuries, and to implement preventative programmes minimizing rates of occupational hazards for paramedics. The authors point out that the system of gathering data on sharp injuries in hospital medical personnel in Poland should be improved. Thus, the gathered data should be urgently evaluated.

## Figures and Tables

**Table 1:** Total number of work-related accidents and sharp injuries in the hospital and in paramedics employed in selected wards, in 2005–2015.

**Year**	**Total Number of Work-Related Accidents in Hospital**	**Number of Accidents in HED**	**Workers Who Have Suffered an Accident in HED**		**Number of Accidents in EMS**	**Workers Who Have Suffered an Accident in EMS**			
			**Paramedic**	**Nurse**		**Paramedic**	**Paramedic Driver**	**Nurse**	
2005	30	2	1	1	1	1	0	0	
2006	41	3	1	2	5	3	2	0	
2007	18	0	0	0	4	1	2	1	
2008	24	2	0	2	0	0	0	0	
2009	21	0	0	0	5	3	1	1	
2010	39	1	0	1	8	6	2	0	
2011	45	1	1	0	5	4	1	0	
2012	37	6	0	6	4	3	1	0	
2013	46	4	0	4	6	5	1	0	
2014	47	3	2	1	4	4	0	0	
2015	42	2	1	1	5 and 1 *	5	2	0	
total	390	24	6	18	48	35	12	2	
**Year**	**Total Number of Injuries in Hospital**	**Number of Injuries in HED**	**Injured Workers in HED**		**Number of Injuries in EMS**	**Injured Workers in EMS**			
			**Paramedic**	**Nurse**		**Paramedic**	**Paramedic Driver**	**Nurse**	**Doctor**
2005	34	3	0	3	4	3	0	1	0
2006	25	3	1	2	2	1	0	1	0
2007	20	3	0	3	7	2	0	5	0
2008	24	4	3	1	6	3	1	2	0
2009	16	3	1	2	3	2	1	0	0
2010	23	4	0	4	4	2	1	1	0
2011	31	3	0	3	4	3	1	0	0
2012	28	3	0	3	2	1	0	0	1
2013	18	4	1	3	3	2	1	0	0
2014	20	1	1	0	2	2	0	0	0
2015	26	2	0	2	2	1	1	0	0
total	265	33	7	26	39	22	6	10	1

* Collective accident; HED—Hospital Emergency Department; EMS—Emergency Medical Service.

**Table 2:** The ratio of accidents in selected wards, the ratio of accidents in which paramedics were involved and the accident rate in paramedics, 2005–2015.

Year	Total Number of Work-Related Accidents	Ratio of Work-Related Accidents in HED to the Total Number of Work-Related Accidents in the Hospital (%)	Ratio of Paramedics Involved in Work-Related Accidents to the Total Number of Employees of the HED Involved in Work-Related Accidents (%)	Ratio of Work-Related Accidents in the EMS to the Total Number of Work-Related Accidents in the Hospital (%)	Ratio of Paramedics Involved in Work-Related Accidents to the Total Number of Employees of the EMS Involved in Work-Related Accidents (%)	Annual Accident Rate per 100 Employed Paramedics
2005	30	6.67	50.00	3.33	100.0	2.79
2006	41	7.32	33.30	12.2	100.0	6.80
2007	18	0.0	0.0	22.22	75.0	3.65
2008	24	8.33	0.0	0.0	0.0	0.0
2009	21	0.0	0.0	23.81	80.0	4.70
2010	39	2.56	0.0	20.51	100.0	9.34
2011	45	2.22	100.0	11.11	100.0	7.00
2012	37	16.22	0.0	10.81	100.0	4.75
2013	46	8.7	0.0	13.04	100.0	6.06
2014	47	6.38	66.67	8.51	100.0	6.12
2015	42	4.76	50.00	16.67	100.0	7.55
Total/Mean	390	6.15		12.31		5.34

HED—Hospital Emergency Department; EMS—Emergency Medical Service.

**Table 3:** Accidents at work among paramedics (including paramedic drivers) by the time of the day and season.

**Time of the Day/Season**	**Accidents Among HED Paramedics**			**Accidents Among EMS Paramedics**		
	**In 2005–2009**	**In 2010–2015**	**Total**	**In 2005–2009**	**In 2010–2015**	**Total**
Morning (7:00 a.m.–12:00 p.m.)	1	1	2	3	6	9
Afternoon (12:00 p.m.–7:00 p.m.)	0	1	1	5	8	13
Evening-night (7:00 p.m.–7:00 a.m.)	1	2	3	5	20	25
	Chi^2^Pearson = 0.75; *p* = 0.69; C-Pearson = 0.33			Chi^2^Pearson = 1.64; *p* = 0.44; C-Pearson = 0.18		
**Season**	**In 2005–2009**	**In 2010–2015**	**Total**	**In 2005–2009**	**In 2010–2015**	**Total**
The winter months (October–March)	1	0	1	7	24	31
The summer months (April–September)	1	4	5	6	10	16
	Chi^2^Pearson = 2.40; *p* = 0.12; C-Pearson = 0.54			Chi^2^Pearson = 1.17; *p* = 0.28; C-Pearson = 0.16		
**Department**	**Time of the day**			**Total**		
	**Morning**	**Afternoon**	**Evening-night**			
HED	2	1	3	6		
EMS	9	13	25	47		
Total	11	14	28			
	Chi^2^Pearson = 0.77; *p* = 0.68; C-Pearson = 0.12					
**Department**	**Season**			**Total**		
	**Winter**		**Summer**			
HED	1		5	6		
EMS	31		16	47		
Total	32		21	53		
	Chi^2^Pearson = 5.40; *p* = 0.02; C-Pearson = 0.30					

Chi Pearson^2^—Chi-square test; *p*—*p*-value; C-Pearson—Pearson’s contingency coefficient.

**Table 4:** Causes of accidents at work among paramedics, 2005–2015.

Accident Cause	In Each Category (n)	Total (N = 53)	
		n	%
Absence or inappropriate use of material agent		1	1.88
Improper capture	1		
Incorrect employee action		36	67.92
Insufficient concentration on the work being done	7		
The surprise of an unexpected event	22		
Improper pace of work	2		
Other (e.g., bad footfall)	5		
Other causes, not listed in the statistical accident card		16	30.2
Patient aggression	1		
The collision with another vehicle	3		
Physical effort/raising the patient	12		

**Table 5:** Sharp injuries in employees of the Hospital Emergency Department and the Emergency Medical Service, 2005–2015.

**Year**	**Total Number of Hospital Injuries**	**Total Number of Injuries in HED**	**Ratio of Injuries in the HED to the Total Number of Injuries (%)**	**Total Number of Injured Paramedics (** **HED)**	**Ratio of Injured Paramedics to the Total Number of Injured Employees of the HED (%)**	**Annual Injury Rate/100 Paramedics (HED)**
2005	34	3	8.82	0	0.0	0.0
2006	25	3	12.00	1	33.33	20.0
2007	20	3	15.00	0	0.0	0.0
2008	24	4	16.67	3	75.00	42.86
2009	16	3	18.75	1	33.33	14.29
2010	23	4	17.39	0	0.0	0.0
2011	31	3	9.68	0	0.0	0.0
2012	28	3	10.71	0	0.0	0.0
2013	18	4	22.22	1	25.00	7.69
2014	20	1	5.00	1	100.00	8.33
2015	26	2	7.69	0	0.0	0.0
**Year**	**Total Number of Hospital Injuries**	**Total n Number of Injuries in EMS**	**Ratio of Injuries in the EMS to the Total Number of Injuries (%)**	Total Number of Injured Paramedics (EMS) *	Ratio of Injured Paramedics to the Total Number of Injured Employees of the EMS (%) *	Annual Injury Rate/100 Paramedics (EMS) *
2005	34	4	11.76	3	75.00	4.50
2006	25	2	8.00	1	50.00	1.20
2007	20	7	35.00	2	28.57	2.62
2008	24	6	25.00	4	66.67	5.18
2009	16	3	18.75	3	100.0	3.84
2010	23	4	17.39	3	75.00	3.81
2011	31	4	12.90	4	100.0	5.08
2012	28	2	7.14	1	50.00	1.29
2013	18	3	16.67	3	100.0	3.49
2014	20	2	10.00	2	100.0	2.33
2015	26	2	7.69	2	100.0	2.33

* including paramedics holding a driving licence; HED—Hospital Emergency Department; EMS—Emergency Medical Service.
